# Digital versus Conventional Impression Taking Focusing on Interdental Areas: A Clinical Trial

**DOI:** 10.3390/ijerph17134725

**Published:** 2020-06-30

**Authors:** Maximiliane Amelie Schlenz, Victoria Schubert, Alexander Schmidt, Bernd Wöstmann, Sabine Ruf, Katharina Klaus

**Affiliations:** 1 Dental Clinic, Department of Prosthodontics, Justus Liebig University, 35392 Giessen, Germany; victoria.schubert@dentist.med.uni-giessen.de (V.S.); alexander.schmidt@dentist.med.uni-giessen.de (A.S.); bernd.woestmann@dentist.med.uni-giessen.de (B.W.); 2 Dental Clinic, Department of Orthodontics, Justus Liebig University, 35392 Giessen, Germany; sabine.ruf@dentist.med.uni-giessen.de (S.R.); katharina.klaus@dentist.med.uni-giessen.de (K.K.)

**Keywords:** intraoral scanners, periodontally compromised dentition, full-arch impression, aligner treatment, orthodontics, digital prosthodontics, clinical trial

## Abstract

Due to the high prevalence of periodontitis, dentists have to face a larger group of patients with periodontally compromised dentitions (PCDs) characterized by pathologic tooth migration and malocclusion. Impression taking in these patients is challenging due to several undercuts and extensive interdental areas (IAs). The aim of this clinical trial was to analyze the ability of analog and digital impression techniques to display the IAs in PCDs. The upper and the lower jaws of 30 patients (*n* = 60, age: 48–87 years) were investigated with one conventional impression (CVI) using polyvinyl siloxane and four digital impressions with intraoral scanners (IOSs), namely True Definition (TRU), Primescan (PRI), CS 3600 (CAR), and TRIOS 3 (TIO). The gypsum models of the CVIs were digitalized using a laboratory scanner. Subsequently, the percentage of the displayed IAs in relation to the absolute IAs was calculated for the five impression techniques in a three-dimensional measuring software. Significant differences were observed among the impression techniques (except between PRI and CAR, *p*-value < 0.05). TRU displayed the highest percentage of IAs, followed by PRI, CAR, TIO, and CVI. The results indicated that the IOSs are superior to CVI regarding the ability to display the IAs in PCDs.

## 1. Introduction

**Figure 1:**
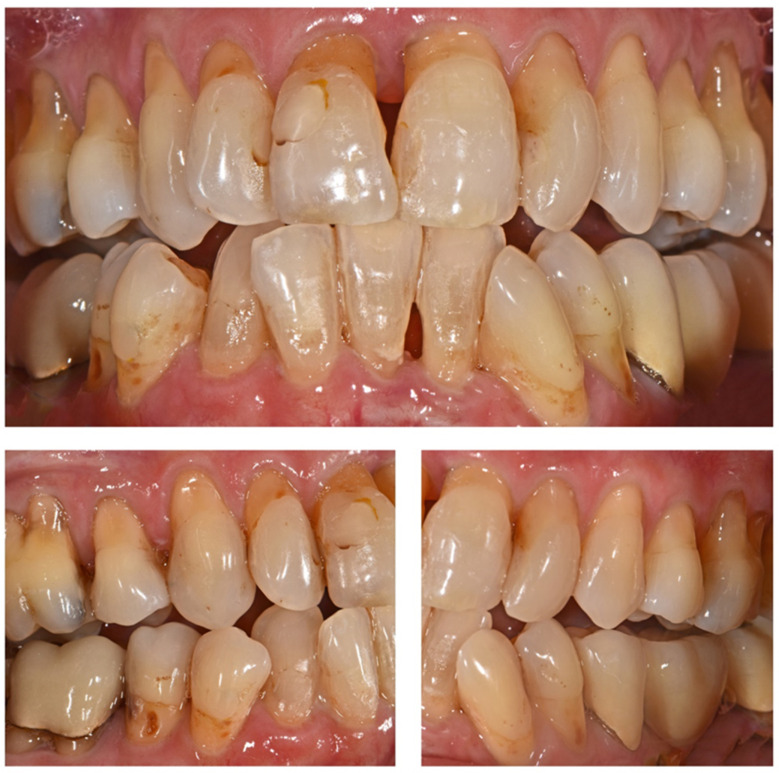
Clinical example of a 65-year-old female patient with a periodontally compromised dentition.

Due to the high prevalence of periodontitis [1], dentists have to face a larger number of patients with periodontally compromised dentitions (PCDs) characterized by several undercuts and extensive interdental areas (IAs). The prevalence of moderate chronic periodontitis ranges between 20 and 42% in patients aged 40–60 years and increases up to 68% in patients aged above 65 years [2,3]. Severe periodontitis affects 10–37% of patients aged 40–60 years and 29–43% of patients aged above 65 years. Severe periodontitis can result in the loss of severely affected teeth due to periodontal destruction [2]. Frequently observed side effects of periodontitis include pathologic tooth migration and development of malocclusion, especially in the anterior jaws which is characterized by flaring and elongation of the teeth, development of diastemas, bite deepening, and crowding of the incisors [4,5,6]. A clinical example of a typical PCD is shown in Figure 1.

After anti-inflammatory periodontal therapy, many patients seek prosthetic as well as orthodontic treatment. The interdisciplinary orthodontic treatment of older adults has been the fastest growing area in orthodontics in the last decade [7]. The main challenges for the orthodontic treatment of PCDs are the need for the application of light forces and anchorage control, the ability for excellent oral hygiene, and the esthetic demands of patients regarding nearly invisible appliances [5,6,7]. Therefore, aligner treatment may be the best solution for these requirements [8,9,10,11].

Due to the undercuts present in the extensive interdental areas (IAs) of periodontally affected patients, obtaining an accurate conventional impression (CVI) is challenging for prosthetic as well as for orthodontic demands. In terms of the CVIs, the logistics of manufacturing the prosthetic restorations as well as the orthodontic aligners require long-term storable and precise impression materials such as polyvinyl siloxanes or polyethers. Due to attachment loss, the elastomeric material flows into the undercuts of the extensive IAs and sets. As the elasticity of the material is lower than the required removal forces, tearing and distortion of the material may be observed during the removal of the impression.

Especially for orthodontic aligner treatment, excellent impressions are required for treatment planning as well as for aligner fabrication [12,13,14,15,16]. At the beginning of the treatment planning workflow, the planning software divides all teeth into segments automatically according to the underlying algorithms. If the impression fails to display the teeth and the IAs sufficiently, a clear distinction between the adjacent teeth cannot be extrapolated by the software algorithm. Therefore, closed IAs would result in inaccurate segmentation, leading to misshapen digital teeth and the proceeding steps of treatment planning and aligner fabrication would be negatively influenced. As a consequence, aligner manufacturers reject impressions and scans of insufficient quality [16]. 

In the last few decades, all fields of dentistry have become increasingly digital. Particularly, due to the continuous development of intraoral scanners (IOSs), impression taking has changed from indirect digitalization of gypsum models using laboratory scanners to direct digitalization of intraoral situations using IOSs [17]. However, the requirements of the accuracy of full-arch scans depend on the indication of the impression. In prosthodontics, discussions in the literature regarding the accuracy and precision of IOSs are controversial. Some authors described CVIs to be more accurate than digital ones [18,19,20], while others have shown equal or even superior accuracies for the IOS compared to the CVI technique [21,22,23,24,25,26,27,28,29]. For orthodontic purposes, most studies described that full-arch intraoral scans meet the requirements of digital orthodontic workflows [30,31,32,33,34]. However, only a few studies have been conducted in vivo [25,33,34].

Thus, we systematically analyzed the ability to display the IAs of a periodontally compromised test model in a former study by comparing conventional polyvinyl siloxane impressions with two intraoral scanning systems. Within the limitations of an in vitro study, it was concluded that IOS, especially the one using the active wavefront sampling technique, displayed the IAs significantly better than CVIs [29].

To date, most of the studies analyzing full-arch digital impressions have been in vitro studies [19,20,21,22,23,24,25,26,27,28,29,31,32]. To overcome the limitations of our in vitro study, the present clinical trial was conducted. This study aimed to compare the ability of one conventional and four digital impression techniques to reproduce the IAs of PCDs. The following null hypotheses were investigated: (a) there is no significant difference among the five impression techniques and (b) there are no significant differences in the dimensions of the IAs with respect to their reproduction in PCDs.

## 2. Materials and Methods 

**Figure 2:**
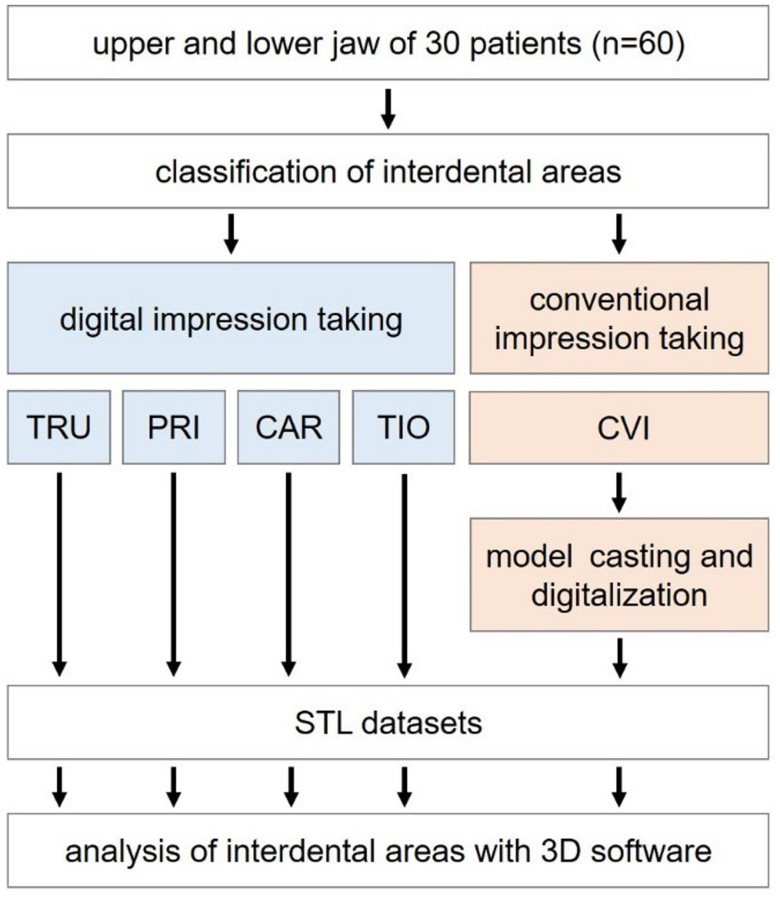
Flow scheme of the clinical trial.

The upper and lower jaws of 30 patients (*n* = 60, 15 females and 15 males, age: 48–87 years) with PCDs undergoing supportive periodontal therapy (SPT) were investigated at the Department of Prosthodontics of the Justus Liebig University Giessen (Germany) between July 2019 and October 2019. The inclusion criteria were ≥10 teeth per jaw and good oral hygiene. Patients with severe systemic diseases or allergies to materials used in the study were excluded. To ensure comparable testing conditions, a single operator (V.S.) experienced in all impression techniques performed the clinical examination. The clinical study was approved by the local ethics committee of the Justus Liebig University Giessen (Ref. no. 71/19). The study was recorded in the German Clinical Trial Register (DRKS00017419). All investigations were performed according to the Declaration of Helsinki. Figure 2 displays the flow scheme of the clinical trial.

**Figure 3:**
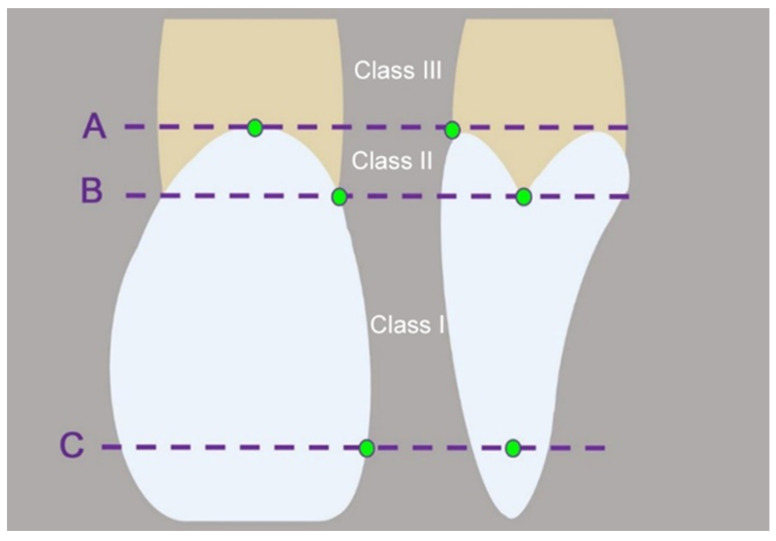
Schematic drawing of the classification system by Nordland and Tarnow [35] to describe the dimension of interdental areas (facial cemento-enamel junction (CEJ, A), interproximal CEJ (B), interdental contact point (C)).

At the beginning of the clinical examination, the dimensions of each IA were classified according to the criteria described by Nordland and Tarnow [35] (Table 1 and Figure 3) [29]. Subsequently, four digital impressions were obtained for each jaw (Table 2). OptraGate (Ivoclar Vivadent, Ellwangen, Germany) was used to retract the cheeks and lips, and dry tips (Microbrush International, Grafton, USA) were placed in the oral cavity to absorb the saliva from the parotid gland. The teeth were gently air-dried. If a calibration device was provided by the manufacturer, it was used to calibrate the tip of the IOS before usage [36]. To ensure a standardized test protocol, the same scanning path was followed for all IOSs beginning with the occlusal surfaces and finishing with the buccal surfaces [37]. Since the intraoral scanner, True Definition (TRU), required a thin layer of titanium dioxide powder (High-Resolution Scanning Spray, 3M, batch-no. NA 28789) for impression taking, digital impressions with Primescan (PRI), CS 3600 (CAR) and TRIOS 3 (TIO) were performed before the TRU digital impression. Data were exported as standard tessellation language (STL) datasets.

For CVIs, dry tips and cheek retractors were removed. A customized metal tray (Ehricke stainless steel, Orbis Dental, Germany) was selected for each jaw and a thin layer of tray adhesive (Universal adhesive, batch no. K010052, Kulzer, Hanau, Germany) was applied. Polyvinyl siloxane impression material (EXA’lence Putty: batch no. 1808131 and Light Body Regular: 1901301, GC, Tokyo, Japan) was used according to the single step putty-wash technique. Before preparing the model with type IV dental stone (Fujirock EP, batch no. 1810031, GC Europe, Leuven, Belgium), impressions were disinfected for 5 min and stored for at least 2 h. Before model casting, CVIs were controlled for torn impression material with gently air drying. In case an exact repositioning was possible (e.g., an interproximal area torn only in the center), the material was repositioned; otherwise the torn material was removed. For standardized procedure, CVIs were only poured once. Before the evaluation, all gypsum models were digitalized with a calibrated high-precision laboratory scanner (ATOS Core, GOM, Braunschweig, Germany) [38].

**Figure 4:**
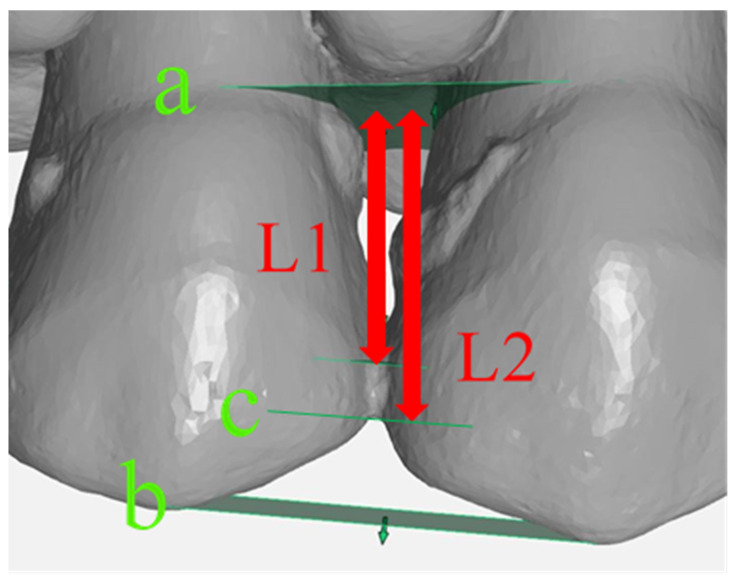
Example of the interdental area (IA) 15/14: Cemento-enamel junction plane (**a**), occlusal plane (**b**), interdental contact point (**c**), displayed IA (L1) in relation to the absolute IA (L2).

For the analysis of the impressions, STL datasets of the four digital impressions and the CVIs were imported to a computer-assisted design software (GOM Inspect 3D, version V8 SR1 2018, GOM, Braunschweig, Germany) and superimposed using a best-fit alignment to ensure the same measurement points for each IA. For standardized measurements, three planes (a, b, c) were constructed for each IA (Figure 4). The interdental contact point was determined 3 mm below the occlusal plane, and the absolute IA was defined as the area between the cementoenamel junction and the interdental contact point. Thus, the absolute IA was identical for the analysis of the different impression techniques. The percentage of the displayed IA in relation to the absolute IA was calculated. This procedure was conducted for each IA.

Statistical analysis was performed using SPSS statistics (version 25, IBM Corp., Armonk, NY USA). The median test was applied, since the data revealed several 0 and 100 values with partial statistical outliers. Finally, p-values were corrected using the Bonferroni method due to the risk of alpha-error accumulation. The level of significance was set at *p*-value < 0.05.

## 3. Results

Altogether, 545 IAs were analyzed. According to the classification by Nordland and Tarnow [35], the following distribution was displayed: 325 class I IAs, 177 class II IAs, and 43 class III IAs. 

**Figure 5:**
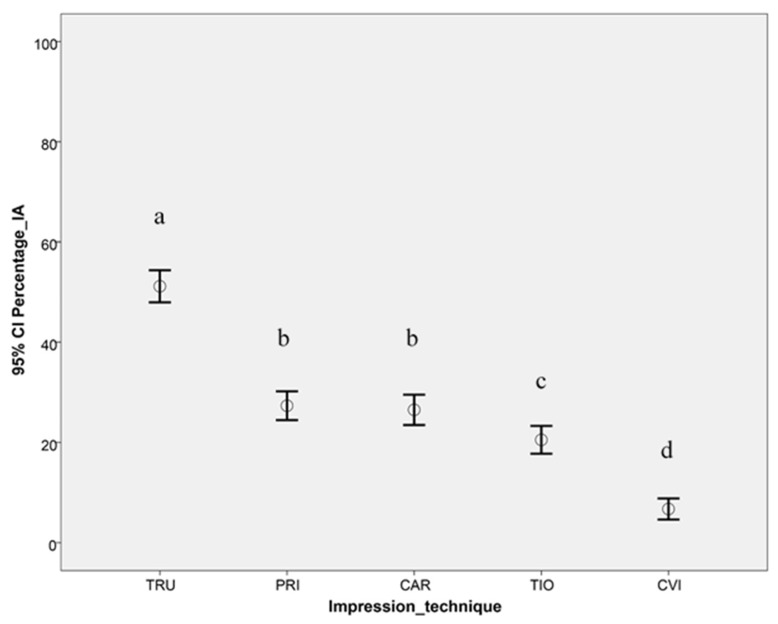
The 95% confidence interval of the displayed interdental area [%] for the five impression techniques (different letters denote significant differences).

The results showed significant differences among different impression techniques (except between PRI and CAR) regardless of the classification (*p*-value < 0.05, Figure 5). Therefore, the first part (a) of the null hypothesis was partially rejected. TRU displayed the highest percentage of IAs, followed by the other digital impression techniques (PRI, CAR, and TIO). CVI showed the lowest percentage of displayed IAs.

**Figure 6:**
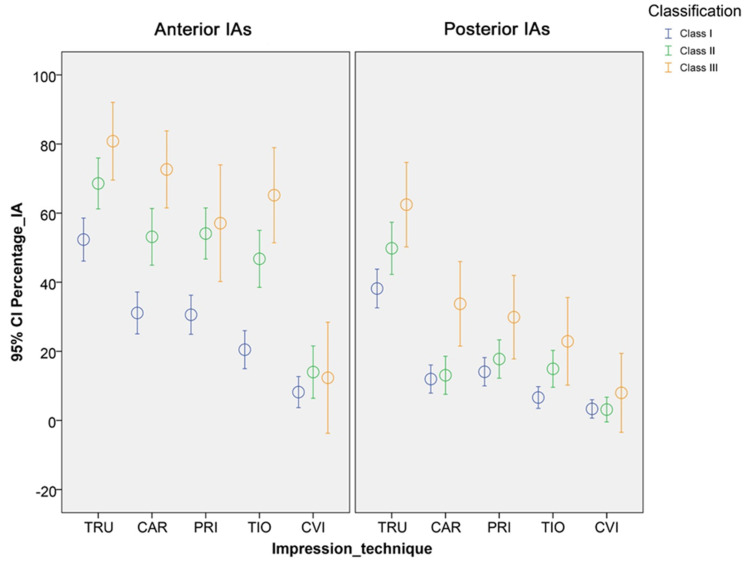
The 95% confidence interval of the displayed interdental area [%] for the different impression techniques and classification by Nordland and Tarnow [35] shown separately for anterior and posterior IAs.

Furthermore, different results were observed for anterior and posterior IAs as well as for the different classifications (Figure 6, Table 3 and Table 4). Particularly, digital impression techniques showed a higher percentage of displayed IAs in the anterior region. Regardless of the impression technique, a tendency toward a higher percentage of displayed IAs was observed for class III IAs when compared with class II and class I IAs. Thus, the second part (b) of the null hypothesis was also partially rejected.

## 4. Discussion

Although many studies have investigated the accuracy of different IOSs for full-arch impressions to date [18,19,20,21,22,23,24,25,26,27,28,29,30,31,32,33,34], most of them have been performed in vitro [19,20,21,22,23,24,26,27,28] and only one in vitro study focused on PCD [29]. To the best of our knowledge, no clinical study has analyzed the ability of different IOS systems to display IAs in PCDs until now. 

The age distribution of patients included in the present study was representative of patients affected by periodontal disease. Additionally, all patients were undergoing SPT with no clinical signs of inflammation and with regular intervals of examination and professional tooth cleaning. Thus, they exhibited PCDs seeking further restorative, prosthetic, or orthodontic treatment for a comprehensive dental rehabilitation. The classes (I–III) according to Nordland and Tarnow [35] were unequally distributed. Class III IAs were especially underrepresented (43 IAs) when compared with classes I and II. This finding might be explained by routine evaluations and an early onset of periodontal diseases in patients. Class III IAs reflect the most severely affected teeth. Additionally, patients must be able to clean their IAs sufficiently. Therefore, some teeth might have been extracted before the study. 

For comparable and standardized measurements, CVIs were indirectly digitalized with a high-precision laboratory scanner [27,39]. The additional step of creating gypsum models and the subsequent indirect digitalization might have led to additional underestimation of the IAs in the CVI group. This can be considered a limitation of the present study. To overcome this, direct digitalization of the CVI would have been beneficial. Nevertheless, the manufacturing of gypsum models from the CVI represents the analog workflow used in dentistry to date. Therefore, the methodology selected in this study points out the differences between conventional and digital impression workflows.

To date, there have been no clinical studies on the accuracy of the scanning path. However, in vitro studies have shown a significant impact of different paths on the accuracy of full-arch scans [37,40]. For a better comparison of different IOS systems, a single recommended scanning path was applied to all IOS systems [37]. Studies have shown that the software version and the calibration of the scanning handpiece can influence the quality of the impressions [36,41]. In the present study, calibration was carried out according to the manufacturer’s specifications. Moreover, the calibration was renewed for each patient. According to the manufacturers, TRU and CAR do not need to be calibrated. To avoid the influence of different software versions, no software updates were performed during the study.

The results of this clinical trial showed a lower percentage of displayed IAs for TRU and TIO when compared with the results of a previous laboratory study wherein the same TRU and TIO hardware were used [29]. This difference can be explained by the presence of clinically influencing factors such as saliva, patient movement, and lack of space due to anatomical limitations [38,42,43,44]. In contrast to a test model, each PCD shows different undercuts, angulations, and distributions of teeth, which might explain the difference in the results between in vitro and in vivo studies. Despite the statistical superiority of the IOS, especially the TRU scanning system, in the present study, the mean values of displayed IAs (Figure 5) were clinically not satisfactory, which must be kept in mind during routine clinical use. The differences when compared with the in vitro results involving PCD [29] underline the necessity of in vivo clinical trials. 

Regardless of the different classes, a higher percentage of displayed IAs was observed in the anterior area than in the posterior area. The anterior area is much more accessible and soft tissues such as tongue, lips, and cheeks are easier to retract. In addition, the salivary flow can be controlled more easily in the anterior areas. Furthermore, a greater angulation of even large scanner handpieces is possible in the anterior areas. This allows easier scanning of the IAs. The anatomy of the anterior teeth, which are narrow and long, often results in a more delicate contact point with a smaller proximal surface. Furthermore, the anterior IAs are not as deep as the posterior IAs due to the oro-vestibular extension of the teeth and the alveolar process. In contrast, the space available in the posterior areas is much more limited. A restricted mouth opening makes accessibility even more difficult. Angulation of the scanner handpiece is possible only to a limited extent, especially in the buccal area. The angle of the light emitted by the optical systems is limited by the angulation of the handpiece. Therefore, undercuts are more difficult or sometimes impossible to record in the focal plane.

A tendency toward greater percentages of displayed IAs in higher classes of Nordland and Tarnow [35] was observed for IOSs. Although the undercuts in class III IAs are deeper than those in class I or II IAs, the neighboring tooth surfaces that have to be captured are at a greater distance from each other. Therefore, the effect of interpolation by the scanning software might have been reduced in larger IAs.

Even though all IOSs displayed a higher percentage of IAs compared to CVI, the results among the IOS groups differed. TIO displayed the lowest percentage of IAs compared to all other IOSs. This finding might be explained by the measuring principle of confocal microscopy. The beam path deflects the reflected light toward the sensor, but the pinhole diaphragm passes only the reflected beams from the object within the focal plane. Moreover, the angulation of the relatively large handpiece is limited, especially in the posterior areas. Due to the measuring principle of the IOS, a larger angulation is necessary to display undercuts, since the beam path is not as straight as the beam path in the other systems.

TRU displayed the highest percentage of IAs using the measuring principle of active wavefront sampling. With this method, areas outside the focal plane can also be captured. However, coating teeth with titanium dioxide powder is required. It creates a higher quality reference pattern [45] and ensures uniform light scattering [46,47]. Ender et al. [45] assumed that coating of the tooth surfaces leads to an improvement in the image, especially at large angulations. Thus, it cannot be ruled out, that the superiority of TRU in the present study must be attributed to the use of powder instead of the differences in measuring principle. Nevertheless, in vivo application of a thin uniform powder layer presents difficulties. Moreover, renewal of coating can lead to inaccuracies and misrepresentations [48,49].

CAR uses the principle of active triangulation with strip-light projection. The elevation profile is captured by a distortion of lines, which makes it difficult to capture a narrow IA. PRI uses a new measuring principle, namely the optical high-frequency contrast analysis, which combines confocal microscopy with strip-light projection [50]. 

In addition to the different measuring principles, the computing algorithm of stitching single images of the scanning process into a three-dimensional image might influence the scanning performance. As long as the IOS manufacturers do not provide any information about the algorithm, its relevance can only be hypothesized.

## 5. Conclusions

In PCD, IOSs and especially TRU, which is based on active wavefront sampling technology, can display a higher percentage of IAs than CVI. The IAs in the anterior area of the jaws are better displayed by the IOSs than the IAs in the posterior area. Additionally, a higher percentage of displayed IAs by IOS was observed for class III IAs according to Nordland and Tarnow [27]. Thus, the use of IOSs in PCDs can be recommended prior to CVI, if displaying the IAs is required. Nevertheless, the correct display of the IAs remains a challenge even in digital impressions.

## Figures and Tables

**Table 1:** Classification system by Nordland and Tarnow [35].

Classification	Description
Class I	The tip of the interdental papilla lies between the interdental contact point and the most coronal extent of the cemento-enamel junction (CEJ) (space present but interproximal CEJ is not visible).
Class II	The tip of the interdental papilla lies at or apical to the interdental CEJ, but coronal to the apical extent of the facial CEJ (interapproximal visible).
Class III	The tip of the interdental papilla lies level with or apical to the facial CEJ.

**Table 2:** Intraoral scanners used in this study.

Abbreviation	Product Name	Software Version	Manufacturer
TRU	True definition	5.1.1	3M (Saint Paul, MN, USA)
PRI	Primescan	5.0.1	Dentsply Sirona (Bensheim, Germany)
CAR	CS 3600	3.1.0	Carestream Dental (Stuttgart, Germany)
TIO	Trios 3 Cart	18.2.10	3Shape (Copenhagen, Denmark)

**Table 3:** Descriptive statistics and results of the Median test corrected by the Bonferroni method for anterior teeth.

Classification	Impression Technique	Descriptive Statistics			*p*-Values (Corrected by Bonferroni)			
		25th Percentile	Median	75th Percentile	PRI	CAR	TIO	CVI
Class I	TRU	0.00	62.26	82.1	<0.001	<0.001	<0.001	<0.001
	PRI	0.00	9.77	55.1	-	1	0.018	<0.001
	CAR	0.00	0.00	59.01	-	-	0.168	<0.001
	TIO	0.00	0.00	44.04	-	-	-	<0.001
	CVI	0.00	0.00	0.00	-	-	-	-
Class II	TRU	62.32	76.2	100.00	<0.001	1	<0.001	<0.001
	PRI	38.58	58.45	74.85	-	1	1	<0.001
	CAR	7.85	58.88	86.30	-	-	1	<0.001
	TIO	0.00	49.68	76.75	-	-	-	<0.001
	CVI	0.00	0.00	0.00	-	-	-	-
Class III	TRU	63.43	87.26	100.00	0.152	1	0.160	<0.001
	PRI	28.50	61.85	84.30	-	1	1	<0.001
	CAR	51.56	73.92	94.92	-	-	1	<0.001
	TIO	42.58	70.34	88.72	-	-	-	<0.001
	CVI	0.00	0.00	0.00	-	-	-	-

**Table 4:** Descriptive statistics and results of the Median test corrected by the Bonferroni method for posterior teeth.

Classification	Impression Technique	Descriptive Statistics			*p*-Value (Corrected by Bonferroni)			
		25th Percentile	Median	75th Percentile	PRI	CAR	TIO	CVI
Class I	TRU	0.00	40.51	72.26	<0.001	<0.001	<0.001	<0.001
	PRI	0.00	0.00	0.00	-	1	0.021	<0.001
	CAR	0.00	0.00	0.00	-	-	1	<0.001
	TIO	0.00	0.00	0.00	-	-	-	0.154
	CVI	0.00	0.00	0.00	-	-	-	-
Class II	TRU	0.00	59.85	78.69	<0.001	<0.001	<0.001	<0.001
	PRI	0.00	0.00	37.44	-	0.667	1	<0.001
	CAR	0.00	0.00	0.00	-	-	1	<0.001
	TIO	0.00	0.00	33.13	-	-	-	<0.001
	CVI	0.00	0.00	0.00	-	-	-	-
Class III	TRU	63.43	87.26	100.00	<0.001	<0.001	<0.001	<0.001
	PRI	28.50	61.85	84.30	-	1	1	<0.001
	CAR	51.56	73.92	94.91	-	-	1	<0.001
	TIO	42.58	70.34	88.72	-	-	-	0.020
	CVI	0.00	0.00	0.00	-	-	-	-
